# An Epidemic of Chronic Pseudophakic Endophthalmitis Due to *Ochrobactrum anthropi*: Clinical Findings and Managements of Nine Consecutive Cases

**DOI:** 10.1080/09273940701798546

**Published:** 2007-12-17

**Authors:** Seung Song, Jae Kyoun Ahn, Gwang Hoon Lee, Yeoung Geol Park

**Affiliations:** Department of Ophthalmology, Chonnam National University Medical School, Gwangju, Korea

**Keywords:** Ochrobactrum anthropi, chronic endophthalmitis, pars plana vitrectomy, partial capsulectomy, antibiotics sensitivity

## Abstract

**Purpose:**

To report an epidemic of *O. anthropi* pseudophakic endophthalmitis.

**Methods:**

The medical records of nine patients with culture-proven *O. anthropi* endophthalmitis were reviewed.

**Results:**

The presenting features were compatible to chronic endophthalmitis. Two patients showed coinfections with *P. acnes*. Antibiotics sensitivity test revealed susceptibility to quinolones. Pars plana vitrectomy (PPV) with partial capsulectomy (PC) cured infections in seven patients without coinfection of *P. acnes*. Final visual acuity was 20/40 or better in five patients.

**Conclusions:**

*O. anthropi* should be considered in cases with chronic pseudophakic endophthalmitis. PPV with PC should be the initial therapeutic option for *O. anthropi* endophthalmitis.

## INTRODUCTION

Chronic postoperative endophthalmitis after cataract surgery has been well known in several small series.^1^^–^7 Because chronic pseudophakic endophthalmitis show the indolent clinical courses and partially respond to steroid therapy, it is particularly difficult to diagnose and treat. The causative organisms to be considered in chronic pseudophakic endophthalmitis are usually *Propionibacterium acnes*, coagulase-negative staphylococci, and rarely fungi.^1^^–^5 Other low-virulent gram-negative pathogens also have been documented to cause a similar clinical entity.^7^^,^^8^ *Ochrobactrum anthropi* is an aerobic, low-virulent, non-lactose-fermenting, gram-negative bacillus that is usually isolated from environmental and hospital water sources.^9^ Of concern in managing patients infected with this organism is its antibiotic resistance to penicillins and cephalosporins.^10^ To date, *O. anthropi* endophthalmitis has been reported in four case reports after intraocular surgery or by endogenous spread.^11^^–^14

In this study, we reported the clinical findings and surgical managements of nine cases with an epidemic of chronic pseudophakic endophthalmitis secondary to *O. anthropi* at the single referring clinic.

## PATIENTS AND METHODS

The medical records of nine patients with the culture-proven *O. anthropi* endophthalmitis referred to us from the single local clinic were reviewed retrospectively. All patients underwent uncomplicated phacoemulsification and implantation of posterior chamber intraocular lens (IOL). Six patients received the surgery on April 3, 2006, and the others on April 4. The following data were collected for each patient: age, gender, presenting clinical features associated with the development of endophthalmitis, interval between surgery and onset of signs and symptoms, results of microbiologic evaluation and culture of ocular specimens, initial treatment method, inflammatory control after therapeutic interventions, subsequent medical or surgical interventions, visual acuity at 1, 3, 6, 12 months, and final follow-up after the curative therapeutic intervention.

All patients underwent a diagnostic vitreous or aqueous biopsy or both to establish the diagnosis of *O. anthropi* endophthalmitis. All ocular specimens were cultured in three general ways: (1) aerobically on blood, chocolate, McConkey, and Sabouraud's agar; (2) anaerobically on blood agar; (3) in thioglycollate medium. To be considered a positive culture, a specimen must demonstrate growth of the same organism on two or more solid culture media or growth on a single medium after identification on an initial smear. Growth judged to be contaminants by microbiology staff was excluded from the current study.

All patients were initially treated at the time of aqueous and vitreous sampling with the intravitreal injection of vancomycin and ceftazidime. However, all patients subsequently underwent pars plana vitrectomy (PPV) with partial posterior capsulectomy (PC) because of the persistent inflammation regardless of intravitreal injections of antibiotics. Each patient who had a recurrence of signs and symptoms associated with endophthalmitis after PPV with PC was also treated at the time of recurrence using repeat PPV, with removal of IOL and residual capsular materials. Pathologic findings in lens capsule and IOL were examined in two patients using scanning electron microscopy (SEM) and transmission electron microscopy (TEM) at the time of repeat PPV. Infections were considered resolved if the patients did not have a recurrence of classic features of endophthalmitis with a minimum follow-up to one year. After complete remission of inflammation over 3 months, scleral fixation of IOL was performed in each patient. All patients were treated by a single surgeon (JKA).

## RESULTS

**FIGURE 1:**
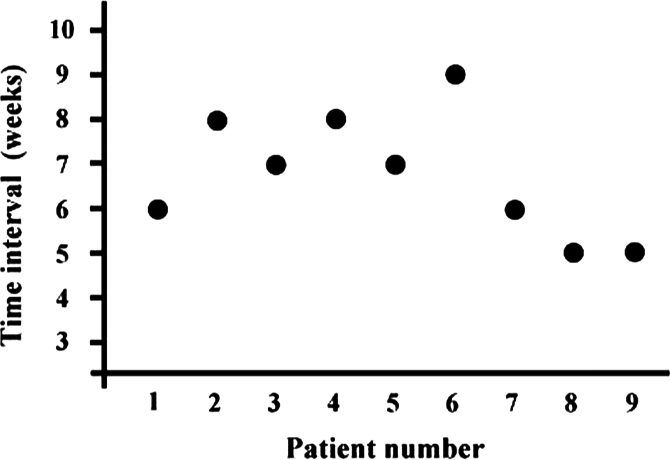
Time interval between cataract surgery and initial treatment of each patient.

**FIGURE 2:**
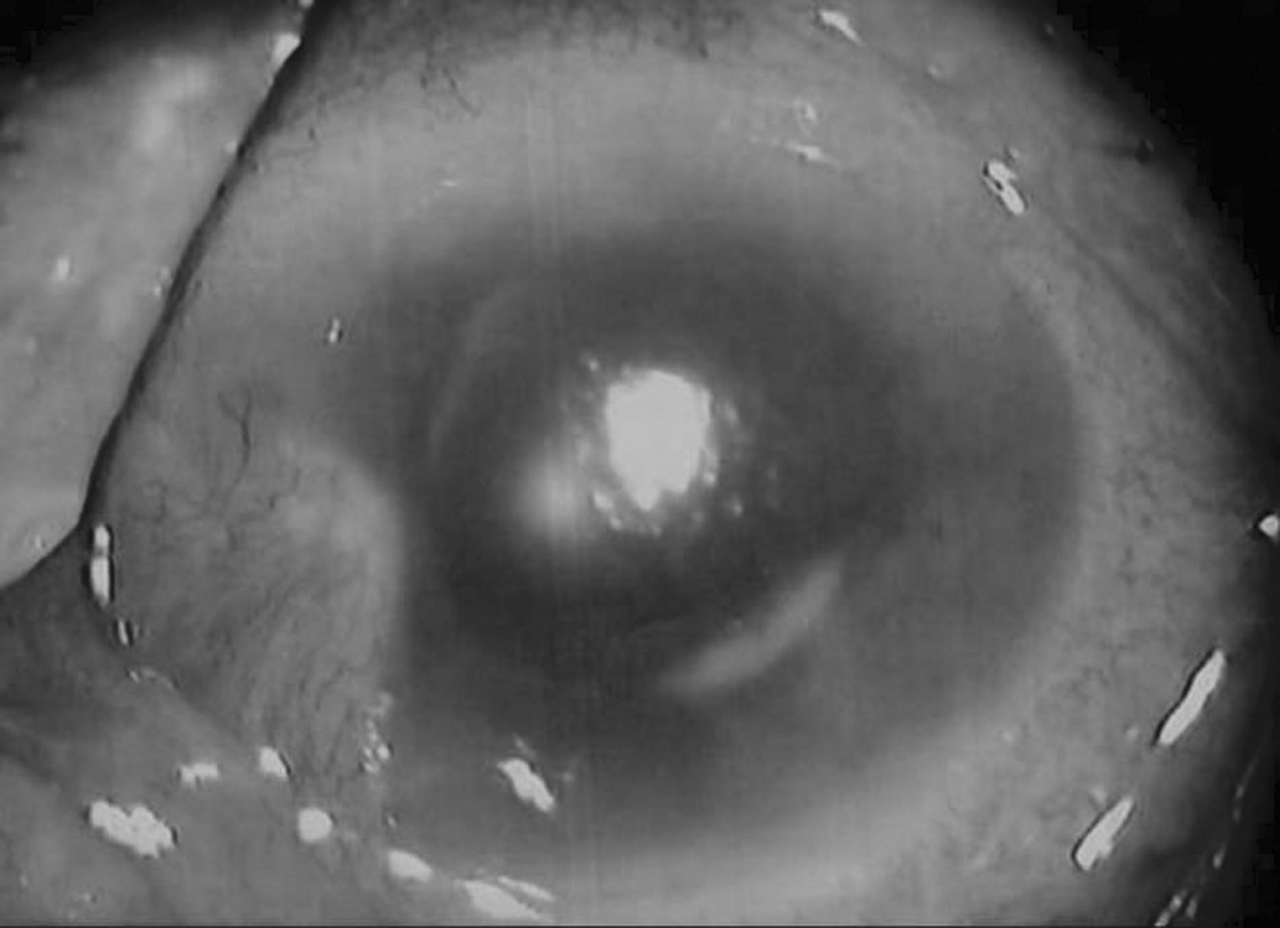
Representative anterior segment photograph of *O. anthropi* endophthalmitis. The photograph shows hypopyon, corneal edema, intraocular fibrin, and capsular white materials.

The average patient age was 68 years (range, 61–79 years) and 2 patients were male. The interval between cataract surgery and initial treatment ranged from 5–9 weeks (mean time to treatment, 6.8 weeks, Figure 1). The presenting features of *O. anthropi* were decreased vision (9/9), anterior chamber reaction (9/9), vitritis (8/9), hypopyon (6/9), and fibrinous pupillary membrane (3/9). Figure 2 illustrates representative findings of anterior segments in patient No. 3 with *O. anthropi* endophthalmitis. The photograph presents granulomatous uveitis with hypopyon, vitritis, and capsular plaque.

Seven of 9 patients had a positive vitreous culture of *O. anthropi* at the initial intravitreal injection of antibiotics. Three cases of 6 patients had a positive aqueous humor culture in addition to a positive vitreous culture. Two patients with the initial culture negative showed a positive culture of *O. anthropi* at the time of therapeutic PPV with PC. The average time to growth of the cultures was 5.5 days for *O. anthropi* (range, 4–6 days). Two cases of 9 patients showed mixed infections with *O. anthropi* and *P. acnes*. The time to growth of the cultures in these patients was 9 and 10 days for *P. acnes*, respectively. The antibiotics sensitivity test of *O. anthropi* resulted in the resistance to β-lactams (ampicillin, amoxicillin, ticarcillin, piperacillin, and ceftazidime) and amino-glycosides (tobramycin and gentamicin), and the susceptibility to quinolones (ciprofloxacin and ofloxacin), trimethoprim-sulfamethoxazole, and imipenem.

**FIGURE 3:**
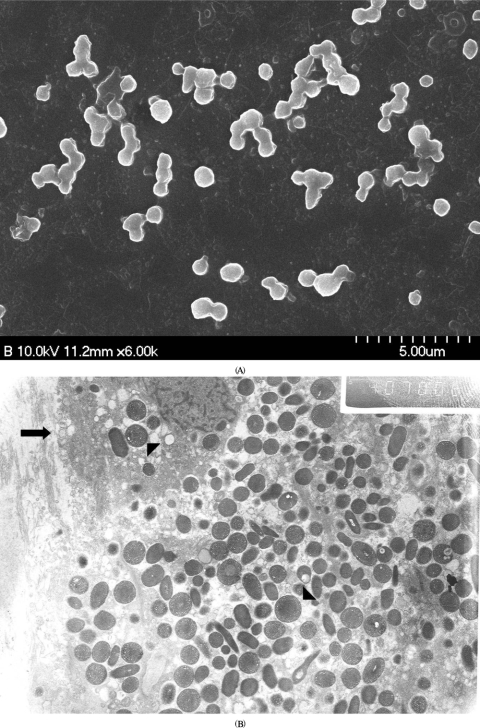
Electron microscopic findings of explanted intraocular lens (IOL) and residual capsule in two patients with coinfection of *O. anthropi* and *P. acnes*. (A) Scanning electron microscopy of a patient (No. 9) reveals polymorphic and rod-shaped bacillus at the surface of explanted IOL. (B) Transmission electron microscopy of a patient (No. 8) also shows the polymorphic organisms with diverse sizes (arrowhead) and a pathogen-laden macrophage (arrow).

Because all patients in our series showed persistent inflammatory signs 2 weeks after initial intravitreal injections of antibiotics, PPV combined with PC were subsequently performed in all patients. Intraocular tamponade by silicone oil was done in 2 patients because of the intraoperative observation of peripheral retinal detachment. These patients received the removal of silicone oil 6 months after PPV with PC. Seven of 9 patients had no recurrences of inflammation after PPV with PC until final follow-up of 14 months. Two of 9 patients did require additional therapy for recurrent inflammation after initial surgery. These patients, especially, showed mixed infections of *O. anthropi* and *P. acnes* at the initial investigation of vitreous culture. Eventual treatment with PPV, total capsulectomy of residual capsular materials, and removal of IOL were performed in these 2 patients, and the explanted IOLs and capsules were examined by electron microscopy. SEM showed polymorphic rod-shaped bacilli (presumed with *P. acnes* and *O. anthropi*) attached to the IOL, consistent with the results of vitreous culture (Figure 3A). As shown in Figure 3B, TEM also revealed the polymorphic organisms and a pathogen-laden macrophage. These patients showed cured infections with no further recurrences after the eventual surgical procedures. Then scleral fixation of IOL was performed in these patients three months after the second surgery. The therapeutic interventions are summarized in Table 1. Overall final visual acuity was 20/40 or better in 5 of 9 patients (56%) and 20/60 or better in all patients after the mean final follow-up of 14 months. Overall visual outcomes are presented in Table 1. The causes of limited visual improvement in the patients with a final vision less than 20/25 were macular degeneration in 3 patients, epiretinal membranes in 3 patients, and persistent macular edema in 1 patient. We did not find any definite abnormality in 2 cases with a final vision of 20/25.

**TABLE 1:** Therapeutic interventions and visual outcomes of patients with *O. anthropi* endophthalmitis

No	Initial V/A	Initial surgery	Recurrence, wks	Second surgery	Recurrence, wks	Tertiary	Final V/A
1	20/200	PPV, PC	Cured	—	—	—	20/25
2	5/400	PPV, PC	Cured	—	—	—	20/40
3	20/400	PPV, PC	Cured	—	—	—	20/30
4	20/400	PPV, PC	Cured	—	—	—	20/40
5	20/400	PPV, PC	Cured	—	—	—	20/25
6	5/400	PPV, PC, SOI	Cured	SOR	—	—	20/50
7	20/400	PPV, PC, SOI	Cured	SOR	—	—	20/60
8	5/400	PPV, PC	7	PPV, TC, IOLR	Cured	SF	20/50
9	5/400	PPV, PC	8	PPV, TC, IOLR	Cured	SF	20/60

V/A = visual acuity; PPV = pars plana vitrectomy; PC = partial posterior capsulectomy; SOI = silicone oil injection; SOR = silicone oil removal; TC = total removal of residual capsular materials; IOLR = intraocular lens removal; SF = scleral fixation of IOL.

## DISCUSSION

Our study demonstrates that *O. anthropi* should be considered to the list of gram-negative organisms associated with chronic pseudophakic endophthalmitis. Furthermore, our results suggest that PPV with PC should be the curative therapeutic option for chronic pseudophakic endophthalmitis associated with *O. anthropi*. However, the presence of polymicrobial infections should be considered in cases that present a recurrence after PPV with PC.

Our study indicated that the clinical presenting signs of *O. anthropi* endophthalmitis were similar to those of chronic endophthalmitis caused by other organisms,^1^^–^8 because *O. anthropi* is a low-virulent gram-negative pathogen. However, the mean time interval between cataract surgery and the initial investigation was relatively shorter in *O. anthropi* endophthalmitis (6.8 weeks) than in *P. acnes* endophthalmitis (36 weeks) or *P. oryzihabitans* (16 weeks) reported by previous studies.^2^^,^^3^^,^^7^ Because average time to growth of the cultures was faster in *O. anthropi* (5.5 days) than in *P. acnes* (9.5 days), the onset of clinical manifestations was faster. One may suspect that our culture results might be false positive; for example, through conjunctiva, but these are unlikely to be false positive because *O. anthropi* has not been implicated in false-positive organisms in the past.

The conventional intravitreal injections of antibiotics (vancomycin and ceftazidime) did not cure *O. anthropi* endophthalmitis because of its innate resistance to antibiotics and sequestration in the capsular bag. In our study, *O. anthropi* was resistant to aminoglycosides and ceftazidime, but sensitive to trimethoprim-sulfamethoxazole and quinolones, consistent with a previous report.^10^ Therefore, our initial intravitreal injection with vancomycin and ceftazidime did not show the therapeutic effect. However, successful treatment of *O. anthropi* endophthalmitis may be possible by injection of sensitive antibiotics simultaneously into the aqueous and vitreous. *O. anthropi* is widely distributed in the water sources, including normal saline and antiseptic solutions.^8^ A contaminated irrigating solution was presumed to be the potential source of *O. anthropi* in our patients.

In this study, PPV with PC was effective to control the *O. anthropi* endophthalmitis in 7 of 9 patients. Initial surgical approach by PPV with PC may offer the theoretical advantages over PPV only or PPV with total capsulectomy and IOL removal. This treatment strategy allows removal of localized infectious sources while leaving enough capsular support to the IOL. It is also attractive because it requires less surgical manipulation and avoids the potentially more serious complications from IOL exchange or removal. In addition, this approach allows for earlier visual rehabilitation and eliminates the need for aphakic correction. However, the remaining 2 patients who had mixed infections of *P. acnes* did require additional therapy because of recurrences. Previous studies also reported that a half of *P. acnes* endophthalmitis did require the additional therapy of total capsulectomy with removal or exchange of IOL after initial treatment of PPV with PC.^2^^,^^3^ Therefore, it may be reasonable to decide on an initial surgical method after the culture report of causative organisms. In patients with chronic endophthalmitis caused by polymicrobial infectious, especially *P. acnes*, removal of the entire capsular bag and the IOL may be performed as a definitive initial therapy and should be performed for recurrent inflammation.

In this study, overall visual outcomes were favorable and comparable to the previous report of chronic endophthalmitis.^1^^–^14 Moreover, 2 patients who had mixed infections of *O. anthropi* and *P. acnes* also showed good visual outcomes after definitive surgical procedures. This indicates that the choice of initial therapy may influence the recurrences of inflammation but does not appear to significantly affect the final visual acuity.

A epidemic of chronic endophthalmitis caused by coagulase-negative staphylococci, *P. acnes*, and fungus have been reported in the literature.^4^^,^^15^ To our best knowledge, this report is the first study to represent a localized outbreak of the largest series of culture-positive chronic pseudophakic endophthalmitis secondary to *O. anthropi*. The initial treatment strategy using PPV combined with partial posterior capsulectomy may offer favorable clinical outcomes regarding inflammatory controls and visual outcomes, but the polymicrobial infections must be suspected in a recurrent case with chronic pseudophakic endophthalmitis. The enthusiastic microbiological investigations hold the key to diagnosis and appropriate management in chronic pseudophakic endophthalmitis.
